# Supplementary material for: An open-source framework for large-scale, flexible evaluation of biomedical text mining systems
Source: J Biomed Discov Collab. 2008 Jan 29;3:1. doi: 10.1186/1747-5333-3-1 (PMC2276192; doi:10.1186/1747-5333-3-1)
Supplement: Additional file 1 — Evaluation platform source code. The additional file contains the software evaluation framework discussed in this paper. The most current release can be downloaded from SourceForge.net [1]. See the accompanying README file for instructions on installation and use. Also included in the distribution is a collection of UIMA wrappers for some commonly used BioNLP tools and annotated corpora. [file 1747-5333-3-1-S1.zip › bionlp_uima_repository-1.0.1/docs/api/src-html/edu/uchsc/ccp/uima/annotation/CCPDocumentSection.html]

```
001    
002    
003    /* First created by JCasGen Tue Aug 28 16:44:10 MDT 2007 */
004    package edu.uchsc.ccp.uima.annotation;
005    
006    import org.apache.uima.jcas.JCas;
007    import org.apache.uima.jcas.JCasRegistry;
008    import org.apache.uima.jcas.cas.FSArray;
009    import org.apache.uima.jcas.cas.TOP_Type;
010    
011    
012    /** The Document Section annotation allows for an entire document section to be annotated as such, and stores section-specific information such as the section identifier.
013     * Updated by JCasGen Tue Aug 28 16:44:10 MDT 2007
014     * XML source: /home/williamb/eclipse/RELEASED_SOURCE_CODE/descriptors/typeSystem/CCPTypeSystem.xml
015     * @generated */
016    public class CCPDocumentSection extends CCPDocumentAnnotation {
017      /** @generated
018       * @ordered 
019       */
020      public final static int typeIndexID = JCasRegistry.register(CCPDocumentSection.class);
021      /** @generated
022       * @ordered 
023       */
024      public final static int type = typeIndexID;
025      /** @generated  */
026      public              int getTypeIndexID() {return typeIndexID;}
027     
028      /** Never called.  Disable default constructor
029       * @generated */
030      protected CCPDocumentSection() {}
031        
032      /** Internal - constructor used by generator 
033       * @generated */
034      public CCPDocumentSection(int addr, TOP_Type type) {
035        super(addr, type);
036        readObject();
037      }
038      
039      /** @generated */
040      public CCPDocumentSection(JCas jcas) {
041        super(jcas);
042        readObject();   
043      } 
044    
045      /** @generated */  
046      public CCPDocumentSection(JCas jcas, int begin, int end) {
047        super(jcas);
048        setBegin(begin);
049        setEnd(end);
050        readObject();
051      }   
052    
053      /** <!-- begin-user-doc -->
054        * Write your own initialization here
055        * <!-- end-user-doc -->
056      @generated modifiable */
057      private void readObject() {}
058         
059     
060        
061      //*--------------*
062      //* Feature: sectionID
063    
064      /** getter for sectionID - gets The section ID for this document section, e.g. 1 = "Title"  2= "Abstract"
065       * @generated */
066      public int getSectionID() {
067        if (CCPDocumentSection_Type.featOkTst && ((CCPDocumentSection_Type)jcasType).casFeat_sectionID == null)
068          jcasType.jcas.throwFeatMissing("sectionID", "edu.uchsc.ccp.uima.annotation.CCPDocumentSection");
069        return jcasType.ll_cas.ll_getIntValue(addr, ((CCPDocumentSection_Type)jcasType).casFeatCode_sectionID);}
070        
071      /** setter for sectionID - sets The section ID for this document section, e.g. 1 = "Title"  2= "Abstract" 
072       * @generated */
073      public void setSectionID(int v) {
074        if (CCPDocumentSection_Type.featOkTst && ((CCPDocumentSection_Type)jcasType).casFeat_sectionID == null)
075          jcasType.jcas.throwFeatMissing("sectionID", "edu.uchsc.ccp.uima.annotation.CCPDocumentSection");
076        jcasType.ll_cas.ll_setIntValue(addr, ((CCPDocumentSection_Type)jcasType).casFeatCode_sectionID, v);}    
077       
078        
079      //*--------------*
080      //* Feature: sectionLength
081    
082      /** getter for sectionLength - gets The length of this document section (in characters).
083       * @generated */
084      public int getSectionLength() {
085        if (CCPDocumentSection_Type.featOkTst && ((CCPDocumentSection_Type)jcasType).casFeat_sectionLength == null)
086          jcasType.jcas.throwFeatMissing("sectionLength", "edu.uchsc.ccp.uima.annotation.CCPDocumentSection");
087        return jcasType.ll_cas.ll_getIntValue(addr, ((CCPDocumentSection_Type)jcasType).casFeatCode_sectionLength);}
088        
089      /** setter for sectionLength - sets The length of this document section (in characters). 
090       * @generated */
091      public void setSectionLength(int v) {
092        if (CCPDocumentSection_Type.featOkTst && ((CCPDocumentSection_Type)jcasType).casFeat_sectionLength == null)
093          jcasType.jcas.throwFeatMissing("sectionLength", "edu.uchsc.ccp.uima.annotation.CCPDocumentSection");
094        jcasType.ll_cas.ll_setIntValue(addr, ((CCPDocumentSection_Type)jcasType).casFeatCode_sectionLength, v);}    
095       
096        
097      //*--------------*
098      //* Feature: sectionName
099    
100      /** getter for sectionName - gets The name of this document section, e.g. Title, Abstract, Introduction, etc.
101       * @generated */
102      public String getSectionName() {
103        if (CCPDocumentSection_Type.featOkTst && ((CCPDocumentSection_Type)jcasType).casFeat_sectionName == null)
104          jcasType.jcas.throwFeatMissing("sectionName", "edu.uchsc.ccp.uima.annotation.CCPDocumentSection");
105        return jcasType.ll_cas.ll_getStringValue(addr, ((CCPDocumentSection_Type)jcasType).casFeatCode_sectionName);}
106        
107      /** setter for sectionName - sets The name of this document section, e.g. Title, Abstract, Introduction, etc. 
108       * @generated */
109      public void setSectionName(String v) {
110        if (CCPDocumentSection_Type.featOkTst && ((CCPDocumentSection_Type)jcasType).casFeat_sectionName == null)
111          jcasType.jcas.throwFeatMissing("sectionName", "edu.uchsc.ccp.uima.annotation.CCPDocumentSection");
112        jcasType.ll_cas.ll_setStringValue(addr, ((CCPDocumentSection_Type)jcasType).casFeatCode_sectionName, v);}    
113       
114        
115      //*--------------*
116      //* Feature: subSections
117    
118      /** getter for subSections - gets Allows for direct linking to CCPDocumentSubSections residing in this document section.
119       * @generated */
120      public FSArray getSubSections() {
121        if (CCPDocumentSection_Type.featOkTst && ((CCPDocumentSection_Type)jcasType).casFeat_subSections == null)
122          jcasType.jcas.throwFeatMissing("subSections", "edu.uchsc.ccp.uima.annotation.CCPDocumentSection");
123        return (FSArray)(jcasType.ll_cas.ll_getFSForRef(jcasType.ll_cas.ll_getRefValue(addr, ((CCPDocumentSection_Type)jcasType).casFeatCode_subSections)));}
124        
125      /** setter for subSections - sets Allows for direct linking to CCPDocumentSubSections residing in this document section. 
126       * @generated */
127      public void setSubSections(FSArray v) {
128        if (CCPDocumentSection_Type.featOkTst && ((CCPDocumentSection_Type)jcasType).casFeat_subSections == null)
129          jcasType.jcas.throwFeatMissing("subSections", "edu.uchsc.ccp.uima.annotation.CCPDocumentSection");
130        jcasType.ll_cas.ll_setRefValue(addr, ((CCPDocumentSection_Type)jcasType).casFeatCode_subSections, jcasType.ll_cas.ll_getFSRef(v));}    
131        
132      /** indexed getter for subSections - gets an indexed value - Allows for direct linking to CCPDocumentSubSections residing in this document section.
133       * @generated */
134      public CCPDocumentSubSection getSubSections(int i) {
135        if (CCPDocumentSection_Type.featOkTst && ((CCPDocumentSection_Type)jcasType).casFeat_subSections == null)
136          jcasType.jcas.throwFeatMissing("subSections", "edu.uchsc.ccp.uima.annotation.CCPDocumentSection");
137        jcasType.jcas.checkArrayBounds(jcasType.ll_cas.ll_getRefValue(addr, ((CCPDocumentSection_Type)jcasType).casFeatCode_subSections), i);
138        return (CCPDocumentSubSection)(jcasType.ll_cas.ll_getFSForRef(jcasType.ll_cas.ll_getRefArrayValue(jcasType.ll_cas.ll_getRefValue(addr, ((CCPDocumentSection_Type)jcasType).casFeatCode_subSections), i)));}
139    
140      /** indexed setter for subSections - sets an indexed value - Allows for direct linking to CCPDocumentSubSections residing in this document section.
141       * @generated */
142      public void setSubSections(int i, CCPDocumentSubSection v) { 
143        if (CCPDocumentSection_Type.featOkTst && ((CCPDocumentSection_Type)jcasType).casFeat_subSections == null)
144          jcasType.jcas.throwFeatMissing("subSections", "edu.uchsc.ccp.uima.annotation.CCPDocumentSection");
145        jcasType.jcas.checkArrayBounds(jcasType.ll_cas.ll_getRefValue(addr, ((CCPDocumentSection_Type)jcasType).casFeatCode_subSections), i);
146        jcasType.ll_cas.ll_setRefArrayValue(jcasType.ll_cas.ll_getRefValue(addr, ((CCPDocumentSection_Type)jcasType).casFeatCode_subSections), i, jcasType.ll_cas.ll_getFSRef(v));}
147      }
148    
149
```
